# A126 SIGNET-RING CELL CARCINOMA OF THE GALLBLADDER: A CASE REPORT OF UNIQUE BRAIN METASTASIS.

**DOI:** 10.1093/jcag/gwae059.126

**Published:** 2025-02-10

**Authors:** Z Alfaraj, A Grin, M Rai

**Affiliations:** Gastro enterology, Queen’s University, Kingston, ON, Canada; Gastro enterology, Queen’s University, Kingston, ON, Canada; Gastro enterology, Queen’s University, Kingston, ON, Canada

## Abstract

**Background:**

Signet-ring cell carcinoma (SRCC) is a rare and aggressive subtype of gallbladder adenocarcinoma, representing 1-3% of all gallbladder cancer cases. Diagnosis is established through histopathological examination, which reveals mucin-filled cells that push the nucleus to the periphery. This characteristic contributes to its aggressive nature, leading to local invasion and distant metastases. Diagnosis can be challenging due to its insidious onset, being detected incidentally after cholecystectomy for conditions, such as cholecystitis or cholelithiasis, or after only metastatic symptoms arise. While the most common metastatic sites are abdominal organs, this report presents a unique case of significant brain metastasis, underscoring its aggressiveness.

**Aims:**

To describe a patient with poorly cohesive SRCC of the gallbladder, which exhibited exceptionally rare brain metastasis.

**Methods:**

Case report and literature review.

**Results:**

A 46-year-old male with no significant medical or surgical history presented with abdominal pain and jaundice lasting one week. An MRCP revealed a large gallbladder mass with multiple liver metastasis. Endoscopic ultrasound confirmed the gallbladder mass, and a fine needle biopsy established the diagnosis of poorly cohesive signet-ring cell carcinoma [Figure 1]. Staging images revealed multiple metastatic large brain lesions with mass effect, confirmed by MRI. A multidisciplinary team, including gastroenterology, medical oncology, and radiation oncology, developed a management plan. Due to the poor prognosis linked to brain metastases, the approach included palliative chemotherapy with cisplatin, gemcitabine, and durvalumab, alongside palliative radiotherapy to the brain and a tapering course of dexamethasone. Following the first cycle of chemotherapy, the patient reported significant relief from abdominal pain.

**Conclusions:**

Treatment of SRCC of the gallbladder has not been well studied due to its rarity. For cases without metastasis, a combination of cholecystectomy and chemotherapy may enhance patient outcomes, though the roles of radiotherapy and immunotherapy remain unclear. Unfortunately, many patients present at advanced stages with metastasis, leading to a poor prognosis. This underscores the necessity for further research to identify risk factors and improve early detection. Additionally, more studies are needed to clarify diagnostic and therapeutic approaches to optimize treatment and improve patient outcomes.

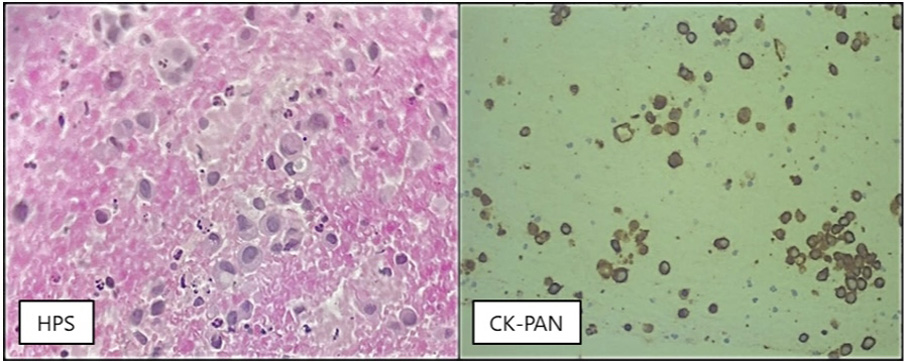

**Funding Agencies:**

**None**

